# Occupational exposure to noise and dust in Swedish soft paper mills and mortality from ischemic heart disease and ischemic stroke: a cohort study

**DOI:** 10.1007/s00420-023-01980-x

**Published:** 2023-06-01

**Authors:** Kjell Torén, Richard L. Neitzel, Helena P. Eriksson, Eva Andersson

**Affiliations:** 1 Occupational and Environmental Medicine, School of Public Health and Community Medicine, Institute of Medicine, Sahlgrenska Academy, University of Gothenburg, Box 414, 405 30 Gothenburg, Sweden; 2 Discipline of Occupational and Environmental Health, University of KwaZulu-Natal, Durban, South Africa; 3 Department of Environmental Health Sciences, University of Michigan, Ann Arbor, MI USA; 4 Department of Occupational and Environmental Medicine, Sahlgrenska University Hospital, Gothenburg, Sweden

**Keywords:** Job-exposure matrix, Physical factors, Occupational health, Cardiovascular diseases, Cerebrovascular diseases

## Abstract

**Objective:**

To elucidate whether occupational noise exposure increases the mortality from ischemic heart disease (IHD) and stroke, and if exposure to paper dust modified the risks.

**Methods:**

We studied 6686 workers from soft paper mills, with occupational noise exposure, < 85 dBA, 85–90 dBA and > 90 dBA, and high (> 5 mg/m^3^) exposure to paper dust. Person-years 1960–2019 were stratified according to gender, age, and calendar-year. Expected numbers of deaths were calculated using the Swedish population as the reference and standardized mortality ratios (SMR) with 95% confidence intervals (95% CI) were assessed.

**Results:**

SMR for IHD was 1.12 (95% CI 0.88–1.41) for noise < 85 dBA, 1.18 (95% CI 0.90–1.55) for 85–90 dBA, and 1.27 (95% CI 1.10–1.47) among workers exposed > 90 dBA. Joint exposure to high noise exposure and high exposure to paper dust resulted in slightly higher IHD mortality (SMR 1.39, 95% CI 1.15–1.67). SMR for ischemic stroke was 0.90 (95% CI 0.37–2.15) for noise < 85 dBA, 1.08 (95% CI 0.45–2.59) for 85–90 dBA, and 1.48 (95% CI 0.99–2.00) among workers exposed > 90 dBA. High noise exposure and high exposure to paper dust resulted in higher ischemic stroke mortality (SMR 1.83, 95% CI 1.12–2.98).

**Conclusion:**

Noise levels > 90 dBA was associated with increased IHD mortality. Combined exposures of noise and paper dust may further increase the risks. Our results do not provide support for a causal relationship for ischemic stroke. Residual confounding from smoking has to be considered. Workers need to be protected from occupational noise levels exceeding 90 dBA.

## Introduction

Noise exposure is common in many workplaces, and hearing loss has been studied in this setting for decades (Lie et al. 2016). There is a growing body of literature indicating that occupational noise exposure also increases the risk of hypertension and ischemic heart disease (Sbihi et al. 2008; Skogstad et al. 2016; Theorell et al. 2016; Kerns et al. 2018; Eriksson et al. 2021). The evidence relating occupational noise exposure and stroke is relatively limited and inconsistent (Fujino et al. 2007; Kolstad et al 2013; Eriksson et al. 2018). Of importance, a recent comprehensive systematic review, including meta-analysis, concluded that there is inadequate evidence for an association between occupational noise exposure ≥ 85 dBA and mortality from ischemic heart disease or stroke (Teixera et al. 2021). The review stressed the need for additional studies estimating the cardiovascular burden, especially mortality, due to occupational noise exposure levels ≥ 85 dBA.

In Sweden, the production of paper is economically important, and one of the main products is soft paper. Soft paper is a semi-manufactured paper product predominantly made with recycled waste paper and used for the production of toilet paper, paper towels, and napkins (Torén et al. 1996). Exposure to high levels of noise in soft paper mills results from both large paper machines as well as small converting machines, where the crude paper is converted to toilet paper, paper towels, and napkins. Soft paper production is labor intensive and women represent a large proportion of the workers. In addition to noise, workers are also exposed to paper dust and shift work (Heederik et al. 1987; Karlsson et al. 2005; Torén et al. 2020). Soft paper dust consists of compressed cellulose fibers, and the physical properties of soft paper dust have been described elsewhere (Sahle et al. 1990).

We have previously analyzed a large cohort of women from six pulp mills and four soft paper mills, finding an increased mortality from myocardial infarction among those with noise exposure exceeding 90 dBA for 10 years or more (Eriksson et al. 2021). As the population was a mixture of pulp mill and different paper mill workers, there was no analysis of the impact of concurrent dust exposure, which differs between these industry sectors, i.e. the dust levels are considerably higher in soft paper mills. Hence, in this paper we present a large cohort of workers, both men and women, only from soft paper mills, with the specific aim to study whether mortality from ischemic heart disease, including myocardial infarction, and ischemic stroke, were increased in relation to occupational noise exposure, and whether occupational paper dust exposure affected these risks.

## Materials and methods

### Study population

This cohort comprises workers from three soft paper mills in Sweden that have previously been described (Torén et al. 2020). Soft paper production started in the oldest mill in 1940, and a few years later the second mill started soft paper production on a small scale. The production of soft paper increased considerably in 1960 in both mills. Both mills previously produced other kinds of paper and also sulphite pulp; this production ended in 1967. In the third mill, soft paper production started in 1947.

The characteristics of the cohort, stratified by gender, are presented in Table 1. We had access to personnel files from all three mills, and all workers newly employed for at least one year between 1960 and 2008 were included in the cohort. From the files we extracted personal data for each cohort member, including the unique personal identification number and employment history (period of employment, department and occupation) at the mill. A total of 8529 workers were available, of which 54 were excluded due to an incomplete personal identification number or lack of employment data. Workers who worked solely in the office (n = 487) or were employed prior to the start of the study in 1960 (n = 1302) were excluded, resulting in a final cohort of 6686 workers potentially exposed to noise, of which 2940 were women.

**Table 1:** Used diagnostic codes in the in the classification of causes of death

Cause of death	International Classification of Diseases (ICD)			
	ICD-7	ICD-8	ICD-9	ICD-10
	1960 to 1968	1969 to 1986	1987 to 1996	From 1997 onwards
Cerebrovascular disease	330–334	430–438	430–438	I60–I69
Ischemic stroke		432–434, 437	433–434	I63
Ischemic heart disease	420, 422.1	410–414	410–414	I20–I25
Myocardial infarction		410	410	I21–I23

### Exposure assessments

We have developed a mill-specific job-exposure matrix (JEM) for noise exposure in soft paper mills for all departments and job titles (Neitzel et al. 2016, 2018). The JEM is based on personal and stationary measurements 1977–2013, as well as information from historical noise maps, and detailed information, including use of hearing protection, collected from focus groups in each soft paper mill. Based on this JEM we categorized each year for workers into one of three categories: < 85 dBA, 85–90 dBA, and > 90 dBA. To facilitate comparisons with other studies we also used a dichotomized definition, < 85 dBA and ≥ 85 dBA, as suggested by the Global Burden of Disease Study (Teixera et al. 2021; Global Burden of Disease 2018).

In all mills, area and personal sampling of total dust was conducted starting in the early 1970s as previously described (Neitzel et al. 2020). We have developed a mill-specific validated JEM for soft paper dust exposure, which was based on expert consensus, work histories, and the results from sampling of total dust (Neitzel et al. 2020, 2022). Based on this JEM, with estimates by job title, department and mill, we assigned each worker a calendar-year specific level of soft paper dust from 1960 until 2008. This allowed us to assess mean exposure to soft paper dust for every year for each worker. In this study we defined “high exposed” workers as those having been exposed to soft paper dust exceeding 5 mg/m^3^ for at least one year, and “low exposed” as < 5 mg/m^3^. This categorization is similar to those used in previous studies of soft paper mills and based on the Swedish threshold limit values for dust at that time (Torén et al. 1994).

### Outcome definitions and follow-up

The start of the follow-up for this mortality study was 1960, or later if the first employment was after 1960. The cohort was followed until death, age of 80 years, or December 31st, 2019, whichever came first. The cohort was matched with the Swedish Cause of Death Register. The period covered the 7th, 8th, 9th, and 10th revisions of the International Classification of Diseases (ICD), and the ICD codes used are displayed in Table 1.

### Statistical analysis

The person-years at risk were calculated and stratified according to gender, age (in 5-year bins), and 1-year calendar periods. The expected numbers of deaths for these strata were calculated using the Swedish population as the reference. Standardized mortality ratios (SMR) were assessed with the assumption of a Poisson distribution of observed deaths, and 95 percent confidence intervals (95% CI) were outlined (Breslow and Day 1987). When there were less than five cases in a group, SMR was not analyzed. The risks assessed as SMR were calculated for three levels of noise exposure; < 85 dBA, 85–90 dBA, and > 90 dBA, as well as for a dichotomized noise exposure variable (< 85 dBA and ≥ 85 dBA). We also present results stratified for gender. In addition, we assessed SMR for the three levels and two levels of noise exposures among two different groups of soft paper dust exposure: high levels (≥ 5 mg/m^3^ for at least one year) and low levels (< 5 mg/m^3^). All analyses were performed using STATA SE 16 (Stata Statistical Software, Release 16) and SAS 9.4 (SAS Institute, Cary, NC, USA).

## Results

The cohort consisted of 6686 workers, and of these 1403 (21.0%) died before 80 years of age during the observation period, 1960–2019. The total number of person-years of follow up was 248,097, of which women comprised 112,889 (45.5%). The group with the highest noise exposure, > 90 dBA included 3885 workers (58.1%), and 5632 workers had noise exposure ≥ 85 dBA (Table 2).

**Table 2:** Descriptive data of the included production workers from three Swedish soft paper mills

	All (N = 6686)	Women (N = 2940)	Men (N = 3746)	Noise exposure < 85 dBA (N = 1054)	Noise exposure 85–90 dBA (N = 1747)	Noise exposure > 90 dBA (N = 3885)
Age at hire, years, mean (SD)	26.8 (11.5)	26.9 (11.4)	26.8 (11.5)	28.4 (12.5)	26.0 (10.4)	26.8 (11.6)
Age at follow-up, years, mean (SD)	66.0 (13.3)	67.8 (12.8)	64.7 (13.5)	66.5 (14.5)	61.2 (13.5)	68.1 (12.2)
Follow-up person-years	248,097	112,889	135,208	37,608	58,512	151,978
Employment time, years, mean (SD)		8.4 (8.7)	10.3 (10.7)	8.4 (9.0)	9.8 (9.4)	9.6 (10.3)
Cumulative paper dust exposure, mg/m^3^ years, mean (SD)	25.6 (36.9)	27.9 (33.9)	23.7 (38.9)	3.7 (7.0)	15.5 (17.4)	36.0 (43.6)
Ever exposed to > 5 mg/m^3^ of paper dust	28.7% (n = 1919)	31.6% (n = 930)	26.4% (n = 989)	0	3.4% (n = 59)	47.9% (n = 1860)

The all-cause mortality was not increased in workers with low noise exposure < 85 dBA, (SMR 0.92, 95% CI 0.82–1.05), or with 85–90 dBA exposure (SMR 1.06, 95% CI 0.94–1.20) (Table 3). The all-cause mortality was increased in workers with > 90 dBA noise exposure (SMR 1.14, 95% CI 1.07–1.22). There was increased mortality for ischemic heart disease and myocardial infarction in workers with noise exposure > 90 dBA (SMR 1.27, 96% CI 1.10–1.47, and SMR 1.47, 95% CI 1.24–1.754, respectively). There was no increased mortality for any of the specific outcomes in workers exposed to noise levels 85–90 dBA or noise levels < 85 dBA, of note, the ischemic stroke mortality in workers with > 90 dBA exposure was 1.48 (95% CI 0.99–2.21).

**Table 3:** Mortality among Swedish soft paper mill workers 1960–2019 by level of noise exposure and gender

Causes of death	Noise exposure < 85 dBA		Noise exposure 85–90 dBA		Noise exposure > 90 dBA		Noise exposure ≥ 85 dBA	
	O	SMR (95% CI)	O	SMR (95% CI)	O	SMR (95% CI)	O	SMR (95% CI)
All N = 6686								
All causes	243	0.92 (0.82–1.05)	260	1.06 (0.94–1.20)	900	1.14 (1.07–1.22)	1160	1.12 (1.06–1.19)
IHD	70	1.12 (0.88–1.41)	51	1.18 (0.90–1.55)	183	1.27 (1.10–1.47)	234	1.25 (1.10–1.42)
Myocardial infarction	45	1.15 (0.86–1.54)	36	1.35 (0.97–1.87)	132	1.47 (1.24–1.75)	168	1.44 (1.24–1.68)
Cerebrovascular disease	17	1.00 (0.62–1.61)	16	1.17 (0.72–1.91)	56	1.14 (0.87–2.21)	72	1.14 (0.91–1.44)
Ischemic stroke	5	0.90 (0.37–2.15)	5	1.08 (0.45–2.59)	24	1.48 (0.99–2.21)	29	1.39 (0.97–2.00)
Men N = 3746								
All causes	214	0.93 (0.81–1.06)	184	1.05 (0.91–1.22)	506	1.15 (1.06–1.26)	690	1.13 (1.04–1.21)
IHD	61	1.06 (0.82–1.36)	44	1.23 (0.92–1.65)	125	1.26 (1.06–1.50)	169	1.25 (1.07–1.46)
Myocardial infarction	38	1.06 (0.77–1.46)	30	1.37 (0.96–1.96)	90	1.48 (1.20–1.82)	120	1.45 (1.21–1.73)
Cerebrovascular disease	16	1.11 (0.68–1.81)	15	1.60 (0.96–2.65)	27	1.07 (0.73–1.56)	42	1.21 (0.89–1.64)
Ischemic stroke	5	1.03 (0.43–2.48)	4	NA	12	1.42 (0.81–2.50)	16	1.37 (0.84–2.24)
Women N = 2940								
All causes	29	0.90 (0.62–1.29)	76	1.08 (0.86–1.35)	394	1.13 (1.03–1.25)	470	1.12 (1.03–1.23)
IHD	9	1.79 (0.93–3.45)	7	0.93 (0.44–1.95)	58	1.29 (1.00–1.67)	65	1.24 (0.97–1.58)
Myocardial infarction	7	2.21 (1.05–4.63)	6	1.25 (0.56–2.79)	42	1.46 (1.08–1.98)	48	1.43 (1.08–1.90)
Cerebro-vascular disease	1	NA	1	NA	29	1.21 (0.84–1.74)	30	1.06 (0.74–1.52)
Ischemic stroke	0	NA	1	NA	12	1.54 (0.88–2.71)	13	1.41 (0.82–2.43)

Standardized mortality ratios (SMR) with 95% confidence interval (CI)

*O* observed number of deaths, *IHD* ischemic heart disease, myocardial infarction from 1969 to 2019, *NA* not applicable due to few cases (< 5)

However, in the gender-stratified analyses a slightly different pattern was found (Table 3). Among men, the pattern was similar as to all workers. Among men, noise exposure > 90 dBA was associated with increased all-cause mortality (SMR 1.15, 95% CI 1.06–1.26), increased IHD mortality (SMR 1.26, 95% CI 1.06–1.50), and increased myocardial infarction mortality (SMR 1.48, 95% CI 1.20–1.82), but no increased ischemic stroke mortality. There was no increased mortality in the other occupational noise groups. Among women, noise exposure > 90 dBA was also associated with increased all-cause mortality (SMR 1.13, 95% CI 1.03–1.25), increased ischemic heart disease mortality (SMR 1.29, 95% CI 1.00–1.67), and increased myocardial infarction mortality (SMR 1.46, 95% CI 1.08–1.98), but no increased ischemic stroke mortality. However, among women there was also in the low noise exposure group (< 85 dBA) increased myocardial mortality (SMR 2.21, 95% CI 1.05–4.63) and an indication towards increased ischemic heart disease mortality (SMR 1.79, 95% CI 0.93–3.45) (Table 3).

When we stratified the analysis according to paper dust exposure, the mortality was increased for all outcomes (ischemic heart disease, myocardial infarction, ischemic stroke) for the combination of high noise exposure (> 90 dBA) and high paper dust exposure > 5 mg/m^3^ (Table 4). However, there was also increased myocardial infarction mortality (SMR 1.36, 95% CI 1.04–1.78) in the group with low paper dust exposure and high noise exposure. The ischemic stroke mortality was increased for the combination of high noise exposure and high dust exposure (SMR 1.83, 95% CI 1.12–2.98), but not in the combination of high noise exposure and low dust exposure and (SMR 1.07, 95% CI 0.53–2.14).

**Table 4:** Mortality among Swedish soft paper mill workers 1960–2019 by level of noise exposure and by high dust exposure

Causes of death	Noise exposure < 85 dBA		Noise exposure 85–90 dBA		Noise exposure > 90 dBA	
	O	SMR (95% CI)	O	SMR (95% CI)	O	SMR (95% CI)
Paper dust exposure > 5 mg/m^3^, N = 1919						
All causes	NA	NA	6	0.74 (0.33–1.65)	518	1.21 (1.11–1.32)
IHD	NA	NA	1	NA	112	1.39 (1.15–1.67)
Myocardial infarction	NA	NA	1	NA	78	1.56 (1.25–1.95)
Cerebrovascular disease	NA	NA	0	NA	38	1.43 (1.04–1.97)
Ischemic stroke	NA	NA	0	NA	16	1.83 (1.12–2.98)
Paper dust exposure ≤ 5 mg/m^3^, N = 4767						
All causes	243	0.92 (0.82–1.05)	254	1.07 (0.95–1.21)	382	1.07 (0.97–1.18)
IHD	70	1.12 (0.88–1.41)	50	1.19 (0.90–1.57)	71	1.12 (0.89–1.42)
Myocardial infarction	45	1.15 (0.86–1.54)	35	1.35 (0.97–1.88)	54	1.36 (1.04–1.78)
Cerebrovascular disease	17	1.00 (0.62–1.61)	16	1.21 (0.74–1.97)	18	0.79 (0.50–1.26)
Ischemic stroke	5	0.89 (0.37–2.15)	5	1.11 (0.46–2.68)	8	1.07 (0.53–2.14)

Standard mortality ratios (SMR) with 95% confidence interval (CI)

*O* observed number of deaths, *IHD* ischemic heart disease; myocardial infarction from 1969 to 2019, *NA* not applicable due to few cases (< 5)

When we dichotomized the noise levels as < 85 dBA and ≥ 85 dBA, there was an increased mortality in workers exposed ≥ 85 dBA, due to ischemic heart disease (SMR 1.25, 95% CI 1.10–1.42), and myocardial infarction (SMR 1.44, 95% CI 1.24–1.68), but not for ischemic stroke (Table 3). The mortality patterns for men and women were similar, except that among the myocardial infarction mortality among women was increased both in the low noise exposure group (< 85 dBA) and the high noise group (≥ 85 dBA).

In the group with noise exposure ≥ 85 dBA and high paper dust exposure > 5 mg/m^3^, there were increased all-cause mortality, increased ischemic heart disease mortality, and increased myocardial infarction mortality (Table 5). The highest mortality was due to ischemic stroke (SMR 1.80, 95% CI 1.10–2.93). When we also added gender stratification, the highest mortality was observed for ischemic stroke among men with high noise exposure and high dust exposure (SMR 1.93, 95% CI 1.04–3.59) (Table 5).

**Table 5:** Mortality among Swedish soft paper mill workers 1960–2019 by level of noise exposure, gender and by high dust exposure

Causes of death	Noise exposure < 85 dBA		Noise exposure > 85 dBA	
	O	SMR (95% CI)	O	SMR (95% CI)
Paper dust exposure > 5 mg/m^3^, all N = 1919				
All causes	NA	NA	524	1.20 (1.10–1.31)
Ischemic heart disease	NA	NA	113	1.38 (1.15–1.66)
Myocardial infarction	NA	NA	79	1.56 (1.25–1.94)
Cerebrovascular disease	NA	NA	38	1.41 (1.02–1.93)
Ischemic stroke	NA	NA	16	1.80 (1.10–2.93)
Paper dust exposure > 5 mg/m^3^, men N = 989				
All causes	NA	NA	314	1.17 (1.05–1.31)
Ischemic heart disease	NA	NA	80	1.33 (1.07–1.65)
Myocardial infarction	NA	NA	54	1.47 (1.12–1.91)
Cerebrovascular disease	NA	NA	22	1.43 (0.94–2.17)
Ischemic stroke	NA	NA	10	1.93 (1.04–3.59)
Paper dust exposure > 5 mg/m^3^, women N = 930				
All causes	NA	NA	210	1.24 (1.09–1.42)
Ischemic heart disease	NA	NA	33	1.52 (1.08–2.14)
Myocardial infarction	NA	NA	25	1.80 (1.22–2.67)
Cerebrovascular disease	NA	NA	16	1.38 (0.84–2.25)
Ischemic stroke	NA	NA	6	1.61 (0.72–3.58)
Paper dust exposure ≤ 5 mg/m^3^, men N = 2757				
All causes	214	0.93 (0.81–1.06)	376	1.09 (0.98–1.20)
Ischemic heart disease	61	1.06 (0.82–1.36)	89	1.19 (0.97–1.47)
Myocardial infarction	38	1.06 (0.77–1.46)	66	1.44 (1.13–1.83)
Cerebrovascular disease	16	1.11 (0.68–1.81)	20	1.03 (0.67–1.60)
Ischemic stroke	5	1.03 (0.43–2.48)	6	0.93 (0.42–2.06)
Paper dust exposure ≤ 5 mg/m^3^, women N = 2010				
All causes	29	0.90 (0.62–1.29)	260	1.04 (0.92–1.18)
Ischemic heart disease	9	1.79 (0.93–3.45)	32	1.05 (0.74–1.48)
Myocardial infarction	7	2.21 (1.05–4.63)	23	1.17 (0.78–1.76)
Cerebrovascular disease	1	NA	14	0.84 (0.50–1.42)
Ischemic stroke	0	NA	7	1.27 (0.61–2.67)

Standard mortality ratios (SMR) with 95% confidence interval (CI)

*O* observed number of deaths, *IHD* ischemic heart disease; myocardial infarction from 1969 to 2019, *NA* not applicable due to few cases (< 5)

## Discussion

An important finding in this large longitudinal cohort study in soft paper workers was that occupational exposure to noise over 90 dBA, as well as exposure to noise ≥ 85 dBA, was associated with increased mortality from ischemic heart disease. There was also an increased myocardial infarction mortality in workers with noise exposure > 90 dBA, but there were increased myocardial infarction mortality in both higher and lower occupational noise levels, which weakens the support of a causal association. The study indicated that the combination of high noise exposure and high paper dust exposure may further increase the mortality, especially regarding ischemic stroke. However, the influence of uncontrolled bias like tobacco smoking is probably of importance.

The finding in the present study addresses a key knowledge gap regarding whether occupational noise exposure increases cardiovascular mortality (Teixera et al. 2021). Previous studies have mostly evaluated the broader outcomes of cardiovascular disease or ischemic heart disease. In addition to ischemic heart disease, we have used the more specific outcome of myocardial infarction. Ischemic heart disease includes, in addition to myocardial infarction, angina pectoris, complications to myocardial infarction and chronic ischemic heart disease. The ischemic heart disease mortality we observed was in general lower compared to myocardial infarction mortality. In the cited review by (Teixera et al. 2021), four cohort studies were identified where ischemic heart mortality was analyzed in relation to occupational noise exposure (Teixera et al. 2021; Gopinath et al. 2011; Suadicin et al. 2012; Pettersson et al. 2020). The pooled effect-estimate in that review indicated only a very small risk increase (RR = 1.03, 95% CI 0.93–1.14) with the range from 0.97 to 1.44 (Teixera et al. 2021). However, this comparison should be judged with caution as the background populations may differ. There was no attempt in that review to separate papers studying myocardial infarction, an outcome that needs further assessment (Troke et al. 2021). Hence, we consider that our study shows an increased ischemic heart mortality among workers occupationally exposed to noise levels exceeding 90 dBA. When we stratified according to gender, we clearly observed increased mortality among men in the high occupational noise group. However, among women the observations were less consistent with increased mortality both in the low and high occupational noise groups. Of note, the power was also low among the women. This may reflect influence of uncontrolled bias, like tobacco smoking.

Regarding cerebrovascular mortality and ischemic stroke mortality, the patterns are less clear.

In published papers, stroke is often defined as ICD10 I60 – I69, or similar codes in the earlier classifications (Gopinath et al. 2011; Pettersson et al. 2020). This is a broad definition that also includes subarachnoid hemorrhage, I60, and transient ischemic attacks, I65. Gopinath et al. did not find any association between occupational noise exposure exceeding five years and mortality due to stroke (Gopinath et al. 2011). Stroke was defined in that study as ICD 10 I60–I69, and the hazard ratio was 1.01, 95% CI 0.62–1.66. In a Swedish study, also with a broad definition of stroke (I60–I69), workers exposed > 85 dBA had an increased stroke mortality, 1.19, 95% CI 1.03–1.38 (Pettersson et al. 2021). For stroke, we have used a more specific definition (I63–I64), ischemic stroke. We did not observe any clearly increased ischemic stroke mortality or cerebrovascular mortality in relation to different groups of occupational noise exposure, even if there was a signal of increased ischemic stroke mortality in workers with > 90 dBA exposure (SMR 1.48, 95% CI 0.99–2.21). Of note, we observed increased ischemic stroke mortality among the subgroup with high occupational noise exposure and high exposure to soft paper dust (SMR 1.83, 95% CI 1.12–2.98). It has been argued that occupational exposure to noise exceeding 80 dBA probably will not increase the risk for stroke (Kolstad et al. 2013; Stokholm et al. 2013). Our results do not provide support for a causal relationship between occupational noise exposure and stroke, as residual confounding such as smoking may be present.

In our main analyses we classified occupational noise into three categories, < 85 dBA, 85–90 dBA and > 90 dBA, as previously recommended (Teixera et al. 2021). That allowed us to look into internal comparisons such as exposure–response relationships. We observed increasing mortality with increasing noise exposure, which may indicate a relationship between occupational noise exposure and all-cause mortality, ischemic heart disease and ischemic stroke. However, the study comprises a long time period when occupational exposures, lifestyle habits including smoking habits, and mortality due to cardiovascular diseases had changed a lot, we applied comparisons with calendar-year specific national mortality rates. Consequently, we did not perform internal comparisons. A variety of mechanisms have been proposed to explain the relationship between noise exposure and cardiovascular diseases. Health effects of noise exposure can be mediated through stimulation of the central stress response, which can result in disturbed sleep, decreased heart rate variability and increase stress hormone levels. Taken together, these changes can directly facilitate the development of cardiovascular disease (Zaman et al. 2022).

Regarding the stratified analyses, the combinations of high noise and high paper dust exposure resulted in a further increased all-cause mortality and further increased mortality from ischemic heart disease, myocardial infarction but also from ischemic stroke. In our previous study we observed among workers with high exposure to soft paper dust an increased cerebrovascular disease mortality (SMR 1.30, 95% CI 1.00–1.67), but no clear increase in all-cause mortality (SMR 1.08, 95% CI 0.99–1.17) or ischemic heart disease (SMR 1.06, 95% CI 0.90–1.26) (Torén et al. 2020). One possibility is that the findings in the present study are the results of an interaction between high noise exposure and dust, as exposure to dust can induce inflammation in the airways, which represents an additional mechanism for cardiovascular disease (Joshi et al. 2022). However, it can also be explained by different smoking habits. In one of the mills we had information about smoking habits, and workers with high exposure to soft paper dust were more often current smokers as compared to low-exposed workers (30% vs 22%). Hence, we consider a possible explanation to the higher mortality, especially from myocardial infarction, in the subgroups with high paper dust exposure could be explained by residual confounding due to tobacco smoking.

Our study has several strengths. Most importantly, we did not rely on self-reported data, but rather used objective data. The exposure assessments were based on validated job-exposure matrices for noise and dust (Neitzel et al. 2016, 2020). The outcomes were also based on causes of death from mortality registers, not on self-reported disease. Another strength was the prospective design with cohort inclusion based on personnel files from the mills which resulted in a cohort that comprised almost all workers that have been employed in these mills, and gave us a comprehensive perspective of soft paper industry employment and exposures. Furthermore, due to our use of personal identification numbers, our medical follow-up had a very high degree of completeness. We also evaluated mortality estimates for men and women separately. A final strength is our simultaneous consideration of paper dust (a chemical hazard) and noise (a physical hazard).

The study has a number of limitations. One limitation is the healthy-worker survivor effect (McMichael 1976). Due to noisy, dusty, and demanding work in paper mills, less healthy workers tend to terminate their employment or be transferred to lower exposed positions in the mills. This can result in weakened associations between noise exposure and outcomes. This bias is likely further accentuated by our use of the total Swedish population as the comparison group, rather than the employed fraction of the population. On the other hand, only newly-employed workers were included in the cohort, which probably diminished healthy-worker survivor effect. Shift work is also a possible confounder, as paper mill workers often have shift work and shift work is clearly linked to increased risk of cardiovascular disease (Torquati et al. 2018). Another important limitation, as mentioned above, is the lack of information on smoking habits for the entire cohort. Since myocardial infarction, ischemic heart disease, and ischemic stroke are associated with smoking, residual confounding was present in our analyses. Another limitation is the existing errors in the applied JEMs, especially regarding the dust JEM for the period before 1970, when exposures were assigned via expert consensus, as no measurement data were available.

In conclusion, we found that occupational noise levels > 90 dBA was associated with increased mortality from ischemic heart disease, but the evidence for an association with myocardial infarction is questionable. Our results do not provide support for a causal relationship between occupational noise exposure and stroke. Workers with joint exposure of high noise levels and high paper dust exposure seemed to have further increased mortality, but that may be due to uncontrolled confounding of tobacco smoking. We have observed similar results for occupational noise exposure ≥ 85 dBA. The results underscore that occupational noise levels must be reduced, and that workers need to be protected to prevent noise-induced hearing loss and to reduce the risk for non-auditory health effects.

## Data Availability

The study outcomes are based on matching with national registers, and the requesters need a Swedish ethical application.
